# Arterial pulsations and transmantle pressure synergetically drive glymphatic flow

**DOI:** 10.1038/s41598-025-97631-x

**Published:** 2025-04-21

**Authors:** Guillermo L. Nozaleda, Wilfried Coenen, Victor Haughton, Antonio L. Sánchez

**Affiliations:** 1 Department of Mechanical and Aerospace Engineering, University of California San Diego, La Jolla, CA 92093-0411 USA; 2 Departamento de Ingeniería Térmica y de Fluidos, Universidad Carlos III de Madrid, 28911 Leganés, Spain; 3 School of Medicine and Public Health, University of Wisconsin-Madison, Madison, WI 53706 USA

**Keywords:** Fluid dynamics, Biological physics, Neuroscience, Biomedical engineering

## Abstract

Clearance of waste material from the brain by the glymphatic system results from net flow of cerebrospinal fluid (CSF) through perivascular spaces surrounding veins and arteries. In periarterial spaces, this bulk flow is directed from the cranial subarachnoid space towards the brain’s interior. The precise pumping mechanism explaining this net inflow remains unclear. While in vivo experiments have shown that the pulsatile motion in periarterial spaces is synchronized with arterial pulsations, peristalsis alone has been deemed insufficient to explain bulk flow. In this study we examine an alternative mechanism based on the interaction between arterial pulsations and fluctuations in transmantle pressure. Previously studied using pressure data from a hydrocephalus patient, this mechanism is analyzed here in healthy subjects using in vivo flow measurements obtained via phase-contrast magnetic resonance imaging. Arterial pulsations are derived from flow-rate measurements of arterial blood entering the cranial cavity, while transmantle-pressure fluctuations are computed using measurements of CSF flow in the cerebral aqueduct. The two synchronized waveforms are integrated into a canonical multi-branch model of the periarterial spaces, yielding a closed-form expression for the bulk flow. The results confirm that the dynamic interactions between arterial pulsations and transmantle pressure are sufficient to generate a positive inflow along periarterial spaces.

## Introduction

**Fig. 1:**
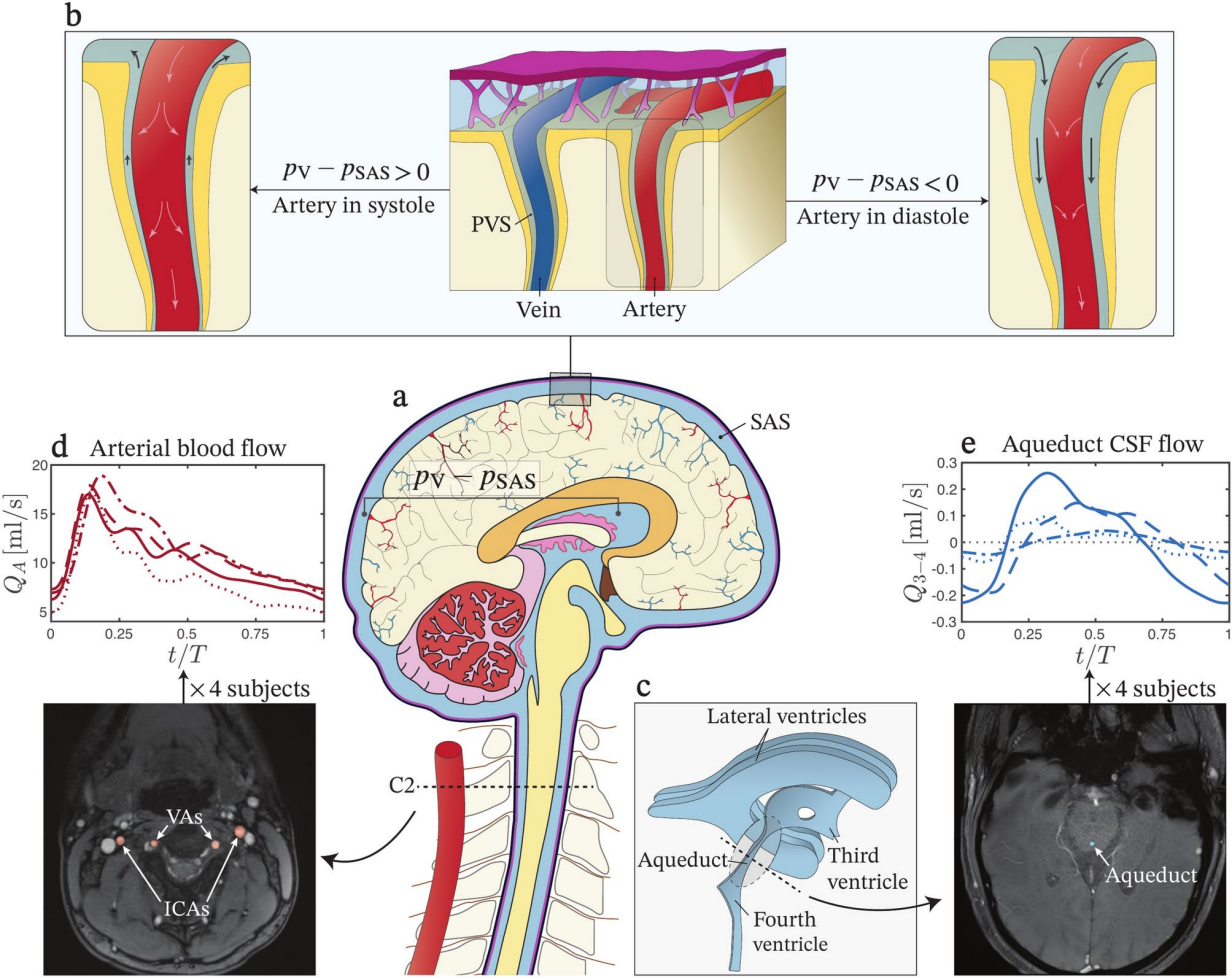
Sketches of the cranial CSF spaces and plots of their cyclic flow patterns. (**a**) Representation of the brain and the primary sites of CSF. (**b**) PVS connecting the cranial SAS with the interior of the brain. Two schematics are included to visualize the dynamic interaction between arterial pulsations and transmantle pressure fluctuations. On the left, resistance to CSF efflux towards the SAS ($p_{\text {V}}-p_{\text {SAS}}>0$) increases during arterial systole. On the right, CSF inflow towards the brain’s interior ($p_{\text {V}}-p_{\text {SAS}}<0$) is facilitated during diastole. (**c**) Representation of the ventricular system, with indication of the cerebral aqueduct that connects the third and fourth ventricle. (**d**, **e**) Simultaneous in vivo MRI measurements in four healthy volunteers of (**d**) arterial blood flow at the C2 level entering the cranial vault (sum of the flow rates in the two vertebral and the two internal carotid arteries), and (**e**) CSF flow rate in the aqueduct measured from the third to the fourth ventricle.

The glymphatic system plays a crucial role in the maintenance of brain health by rinsing waste^1^. Disruptions in glymphatic function have been associated with neurological conditions such as Alzheimer’s and Parkinson’s disease^2–5^. This system, most active during sleep, involves bulk motion of cerebrospinal fluid (CSF) through perivascular spaces (PVS) surrounding veins and arteries^6–8^ (Fig. 1a,b). In periarterial spaces, bulk motion of CSF is directed periodically towards the interior of the brain. From there, CSF enters the brain parenchyma and interchanges with interstitial fluid, facilitating the transport of waste products from the interstitial spaces^9^. Outflow occurs through perivenous spaces, which direct the fluid toward the surface of the brain, where it ultimately exits the central nervous system through lymphatic vessels in the meninges and soft tissues around the skull^10–12^.

Despite the physiological significance of flow in the glymphatic system, the physical mechanisms governing its bidirectional motion remain poorly understood. A plausible explanation for net flow could be the presence of a favorable periodic pressure gradient. In this context, the transmantle pressure—the instantaneous pressure difference between the lateral ventricles and the subarachnoid space (SAS)—has often been used as a proxy for the pressure difference within the PVS^13,14^. Its value fluctuates periodically following the cardiac and respiratory cycles about a small positive average value (i.e. the pressure on average is slightly higher inside the brain). This small overpressure is responsible for the gradual evacuation of CSF produced in the lateral ventricles (Fig. 1c), driving its flow through the foramina of Monro into the third ventricle, then through the cerebral aqueduct into the fourth ventricle, and eventually into the SAS via the foramen of Magendie medially and the foramen of Luschka laterally. Clearly, the small average pressure gradient associated with the cycle-averaged transmantle pressure can explain the bulk motion of CSF towards the exterior of the brain along perivenous spaces. However, in periarterial spaces, the bulk motion is directed inward, opposing this time-averaged pressure gradient,indicating that an additional pumping mechanism is required to overcome the adverse pressure gradient.

As clearly demonstrated in in vivo experiments in mice^9,11^, the movement of fluid in periarterial spaces is synchronized with the arterial wall pulsations. This observation led many authors to hypothesize that periarterial bulk motion is related to peristalsis^15–19^, motivating numerical and analytical work on peristaltic motion in non-axisymmetric annular tubes^20,21^ and multi-branch networks^22^. This perspective has been challenged by many studies^23–26^, which suggest that cardiac-driven peristalsis alone cannot explain CSF bulk motion. The primary criticism is that peristaltic pumping becomes very inefficient when the wavelength of the distension wave is much larger than the length of the channel, that being the case in the periarterial system^27,28^, for which the cardiac-driven arterial wall motion, with characteristic wave lengths exceeding a meter^29^, is better characterized as a synchronous squeeze flow rather than a propagating wave. Alternative explanations for CSF bulk motion include astrocyte endfeet acting as one-way valves^30,31^ and vasomotion, which involves spontaneous or stimulus-evoked changes in vascular diameter which propagate as small-wavelength waves^22,32–34^. Consensus on the precise mechanism connecting the fast, one-second wall pulsations due to the heartbeat to the slow, time-averaged bulk motion remains elusive.

In this paper, we investigate whether the temporal interaction between arterial pulsations and transmantle-pressure fluctuations—previously suggested to influence perivascular flow in the spinal cord^35^—can generate a strong enough pumping mechanism to drive fluid through cerebral periarterial spaces against the existing time-averaged pressure gradient. The central hypothesis is that the timing between transmantle pressure fluctuations and arterial wall pulsations is such that, during each cycle, when the flow is directed outward (toward the SAS), arterial dilation constricts the perivascular space (PVS), increasing flow resistance due to viscous stresses and reducing the outward flow rate (left panel of Fig. 1b). Conversely, half a cycle later, when flow is directed inward, arterial contraction widens the PVS, decreasing flow resistance and enhancing the inflow rate (right panel of Fig. 1b). This dynamic pumping can be interpreted as a streaming flow rate arising in elastic tubes from the deformation of the bounding wall^36^, which has been shown to play a significant role in the CSF bulk motion in the spinal canal^37,38^.

Martinac et al.^39^ evaluated this pumping mechanism in a patient with hydrocephalus, using pressure data from Kasprowicz et al.^40^ and arterial displacement data from Bilston et al^35^. Although they found the mechanism to be largely ineffective, their findings cannot be generalized to healthy individuals, as hydrocephalus patients exhibit abnormal transmantle pressure dynamics^41,42^ and impaired periarterial flow^43^. Therefore, reassessing this mechanism in healthy subjects is of clear interest, and this is the aim of the present investigation.

In this study, we employ in vivo phase contrast (PC) magnetic resonance imaging (MRI) to assess the validity of the pumping mechanism in four healthy volunteers. As detailed below, measurements of arterial blood flow (Fig. 1d) are used to determine arterial pulsation, while measurements of CSF flow in the cerebral aqueduct (Fig. 1e) are used to calculate transmantle pressure fluctuations. Unlike the previous analysis^39^, our data are synchronized with the cardiac cycle, enabling precise determination of the phase offset between the two signals. The two waveforms are then integrated into a multi-branch model of the periarterial system to provide quantitative predictions of perivascular bulk motion. Our results show that, under physiological conditions, the interaction between arterial pulsations and transmantle pressure fluctuations can generate a force strong enough to drive antegrade CSF flow (i.e. in the direction of blood flow) through periarterial spaces.

## Methods

### Evaluation of the transmantle pressure and arterial wall displacement

The periarterial spaces around the cerebral arterial tree constitute a complex branched network stretching from the SAS to the interior of the brain (Fig. 1a,b). The CSF filling the periarterial spaces moves in a pulsating fashion under the simultaneous action of the pressure gradient associated with the transmantle pressure difference and the pulsations of the arterial wall. Predictions of the resulting net flow rate depend critically on the magnitude and phase difference of these two driving mechanisms^39^. The rationale followed in the present analysis for determining the waveforms of these two periodic signals and their relative offset is presented below.

#### The transmantle pressure

The transmantle pressure is the difference between the instantaneous values of the pressure in the lateral ventricles, $p_{\text {V}}( t )$, and that in the cranial subarachnoid space, $p_{\text {SAS}}( t )$. Its value,

1 $$\begin{aligned} p_{\text {V}}-p_{\text {SAS}} = \Delta p_{s} + \Pi ( t ) , \end{aligned}$$

can be expressed as the sum of its cycle-averaged component $\Delta p_{s}=\langle p_{\text {V}}-p_{\text {SAS}} \rangle$, where $\langle \cdot \rangle =T^{-1} \int _t^{t+T} \cdot\,{\textrm{d}}t$ is the time-average operator with $T$ denoting the period of the cardiac cycle, and its fluctuating, time-periodic component $\Pi ( t )=(p_{\text {V}}-p_{\text {SAS}})-\Delta p_{s}$, which has zero mean $\langle \Pi \rangle =0$. Direct measurement of $p_{\text {V}}-p_{\text {SAS}}$ requires the use of dual pressure sensors placed surgically in the cranial cavity, an invasive procedure that is only used when it has clinical potential, e.g. in triaging patients with normal pressure hydrocephalus^44^. The pressure waveform in these patients is highly altered, so that the information acquired has limited value in assessing influences of transmantle pressure difference on periarterial flow in healthy subjects.

In the absence of direct measurements, the values of $p_{\text {V}}-p_{\text {SAS}}$ in asymptomatic individuals must be evaluated indirectly. As reasoned by Jacobson et al.^45,46^, since most of the pressure drop in the ventricular system occurs as CSF flows between the third and fourth ventricles through the narrow cerebral aqueduct (Fig. 1c), the transmantle pressure can be inferred from in vivo measurements of the flow rate $Q_{3{-}4} ( t )$ crossing the aqueduct, which can be acquired using PC-MRI. This rate includes a small cycle-averaged component $q_0=\langle Q_{3{-}4} \rangle$, corresponding to the continuous flow of CSF secreted in the choroid plexus of the ventricles, and a much larger oscillatory component, with peak values that are about two orders of magnitude larger than $q_0$. The noninvasive quantification of the mean flow rate $q_0$ is hindered by the small value of the associated velocities. As a result, while the accuracy of PC-MRI suffices to characterize the CSF oscillatory component, it is however unable to provide reliable measurements of the time-averaged value $q_0$. For the latter, the following analysis will use the value estimated using available physiological information, as described below.

The analysis begins by expressing the aqueduct flow rate $Q_{3{-}4}$ in the Fourier form

2 $$\begin{aligned} Q_{3{-}4}( t ) = \textrm{Re}\left[ \sum _{n=0}^{\infty }q_n\textrm{e}^{\textrm{i}n \omega t}\right] , \end{aligned}$$

where $\textrm{Re}[\cdot ]$ denotes the real part and $\omega =2\pi /T$ is the angular frequency. While the net flow rate $q_0$ is a real number, the coefficients $q_1$, $q_2$, …, characterizing the magnitude and phase of each oscillatory mode, are complex constants. Their values in four different healthy subjects were determined from the Fourier expansion of their waveforms $Q_{3{-}4}( t )$, shown in Fig. 1e, which were acquired using cardiac-gated PC-MRI (details of the measurements are provided below in a separate subsection). As for $q_0$, its estimate takes into account that the CSF secretion rate in humans is approximately 350 $\upmu$l/min^47^. Although different production mechanisms have been proposed in the literature^48,49^, it is widely accepted that about 80% of the total CSF production occurs in the choroid plexi within the cerebral ventricles^1,50,51^, with only 10% taking place in the fourth ventricle^52,53^, so that the net rate crossing the aqueduct can be estimated to be $q_0= 350 \times 0.8 \times 0.9 \simeq 250$ $\upmu$l/min (i.e. $q_0 \simeq 4.2 \times 10^{-3}$ ml/s).

As demonstrated by Sincomb et al.^54^, a sufficiently accurate model to relate $Q_{3{-}4} ( t )$ to $p_{\text {V}}-p_{\text {SAS}}$, first proposed by Bardan et al.^55^, involves the assumption of fully developed unidirectional flow, with the aqueduct geometry approximated as a circular duct of radius *a* and length $L_{3{-}4}$. In this linear approximation, the bulk flow in the aqueduct, $q_0$, and the cycle-averaged transmantle pressure difference, $\Delta p _{s}$, are related by the Hagen–Poiseuille solution, yielding

3 $$\begin{aligned} \Delta p _{s} \simeq \frac{8\rho \nu q_0 L_{3{-}4}}{\pi a^{4}}. \end{aligned}$$

Evaluating the above expression with the density and kinematic viscosity of CSF, $\rho \simeq 10^{3}$ kg/$\hbox {m}^3$ and $\nu \simeq 0.7 \times 10^{-6}$ $\hbox {m}^2$/s, for an aqueduct with $a = 1.5$ mm, $L_{3{-}4}=10$ mm yields $\Delta p _{s}=0.015$ Pa. At the same level of approximation, the fluctuating transmantle pressure $\Pi ( t )$ can be computed in terms of the complex coefficients $q_n$ for $n \ge 1$ with use of the Womersley solution

4 $$\begin{aligned} \Pi ( t ) = \textrm{Re}\left[ \textrm{i} \frac{\rho \omega L_{3{-}4}}{\pi a^2} \sum _{n=1}^{\infty }nq_n\textrm{e}^{\textrm{i}\omega nt} \left( \frac{\beta _n \, J_0\left( \beta _n\right) }{\beta _n \, J_0\left( \beta _n\right) -2 \, J_1\left( \beta _n\right) }\right) \right] ,\quad \text {with} \quad \beta _n=\frac{\textrm{i}-1}{\sqrt{2}}\left( \frac{n \omega a^2}{\nu }\right) ^{1/2}, \end{aligned}$$

where $J_0$ and $J_1$ are the Bessel functions of order 0 and 1. The value of $\Pi ( t )$, calculated from Eq. (4) for $a = 1.5$ mm, $L_{3{-}4}=10$ mm and $\omega =2\pi$ $\hbox {s}^{-1}$ using the first 10 modes $q_1$, …, $q_{10}$, is added to $\Delta p_{s}$ to determine the transmantle pressure $p_{\text {V}}-p_{\text {SAS}}$ for each subject. Figure 3b, to be discussed later in the Results, shows the $\Pi$ functions corresponding to the aqueductal flow rate measurements in Fig. 1e. The values, in the range of a few Pascals, align with the maximal transmantle pressure differences of up to 10 Pa predicted for healthy subjects by Lininger et al.^56^.

#### The arterial pulsations

**Fig. 2:**
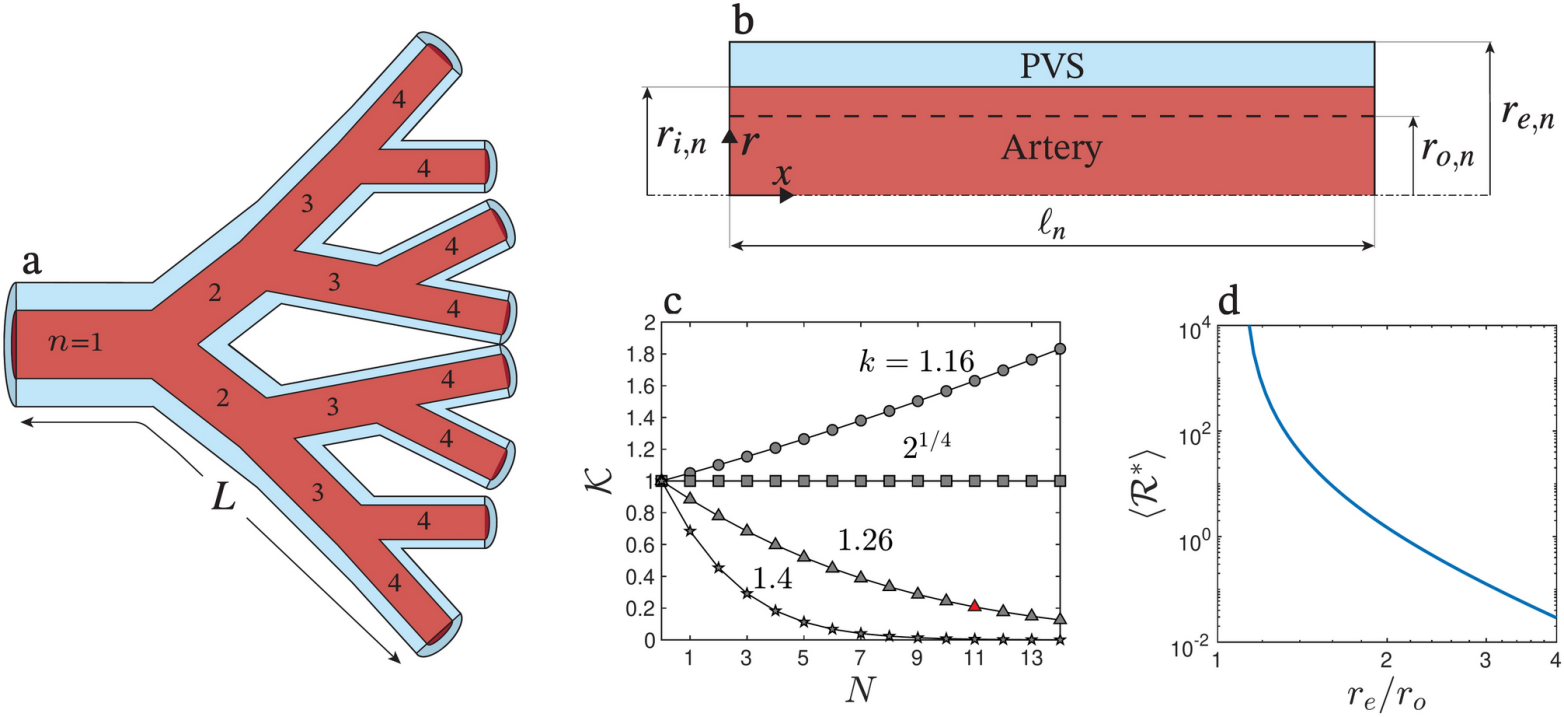
Model of the multi-branched periarterial network. (**a**) Schematic representation of a perivascular tree with an end-to-end length $L$, where each path connecting the cerebral subarachnoid space (SAS) to the brain’s interior undergoes $N=3$ bifurcations. (**b**) Model of the $n$-th element of the network. (**c**) Values of the parameter ${\mathscr {K}}$, which measures the effect of the multi-branched model on bulk flow, obtained for different ratios $k=r_{o,n}/r_{o,n+1}$ as a function of the number of bifurcations $N$. (**d**) Variation of the cycle-averaged dimensionless resistance $\langle {\mathscr {R}}^{*}\rangle$ with $r_e/r_o$ for $\varepsilon =0.1$.

**Fig. 3:**
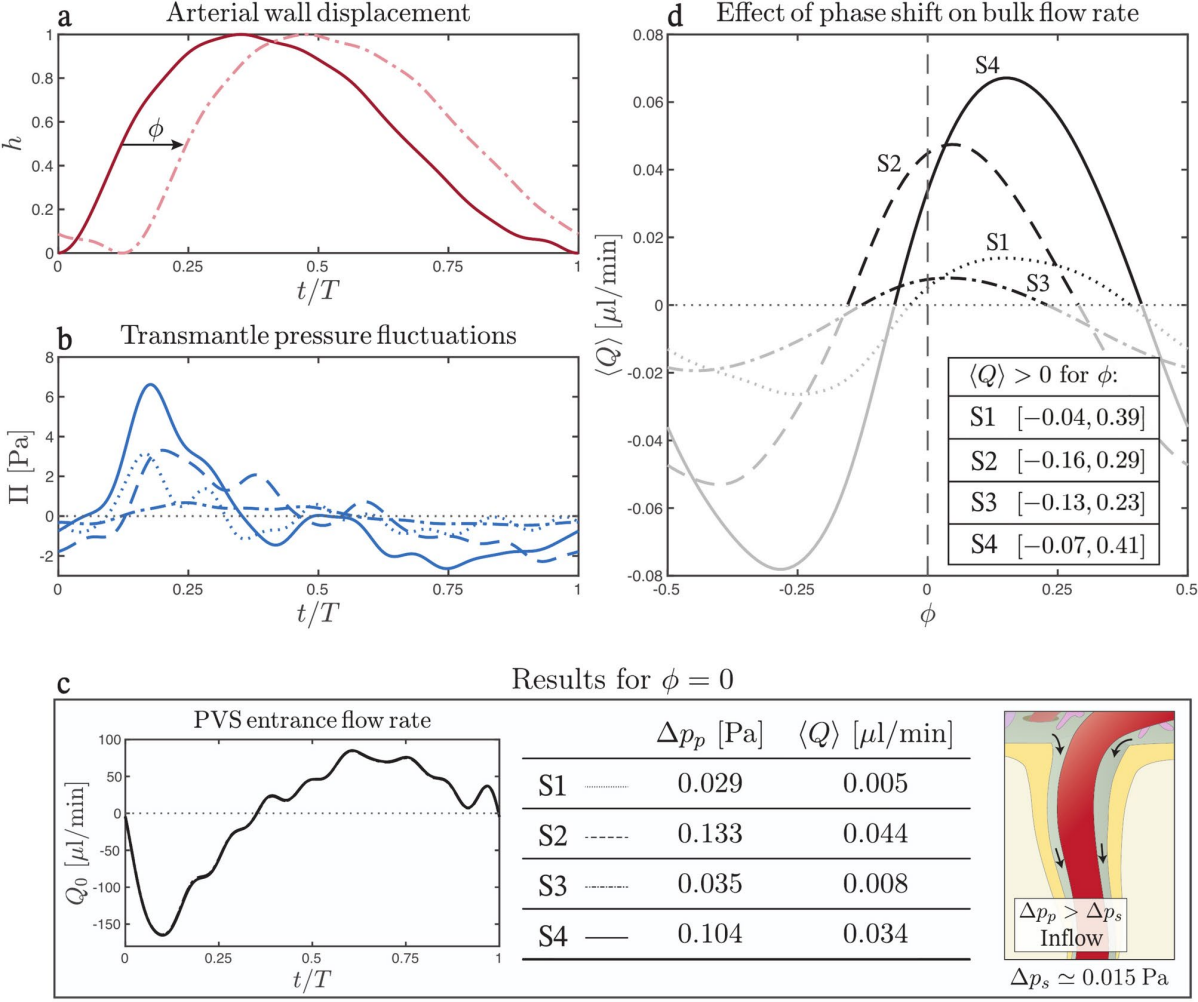
Computed results for each of the four subjects (S1, S2, S3, S4) using the parameters $r_{o}=200$ $\upmu$m,$r_{e}/r_{o}=2$, $L=5$ cm, $\varepsilon =0.1$, $N=11$, and $k=1.26$. (**a**) Normalized arterial wall displacement adapted from Bilston et al.^35^. (**b**) Computed transmantle pressure for the four subjects, corresponding to the aqueductal flow rate measurements shown in Fig. 1e. Both (**a**) and (**b**) are synchronized with the heartbeat, with $\phi$ indicating a small phase offset. (**c**) Results for $\phi =0$, showing the evolution with time of the PVS entrance flow rate $Q_0$ (left) and subject-specific values of the bulk flow rate ($\langle Q\rangle$) and apparent pressure difference ($\Delta p_p$) (right). In all cases, as revealed in the schematic inset, $\Delta p_p$ is greater than $\Delta p_s$, the latter denoting the cycle-averaged transmantle pressure. (**d**) Variation of bulk flows calculated in (**c**) as a function of the phase shift $\phi$, with a table showing, for each subject, the ranges of $\phi$ where bulk inflow of CSF occurs (i.e. $\langle Q \rangle > 0$).

As indicated in Fig. 2b, each element of the perivascular network (i.e. the PVS surrounding an arterial branch between consecutive bifurcations) is represented as an annular region bounded externally by the surface of the brain parenchyma and internally by the circular arterial wall, of radius $r_{i,n}$. In the following description, the external radius of the PVS, $r_{e,n}$, will be taken to be constant, thereby disregarding the small parenchymal deformations that occur at the cardiac frequency^57^. In addition, since the arterial pulse wavelength is much larger than the length of the perivascular element, we shall assume that the arterial deformation is uniform, as described by

5 $$\begin{aligned} r_{i,n}( t ) = r_{o,n} \left[ 1+\varepsilon h ( t ) \right] , \end{aligned}$$

where $r_{o,n}$ is the unperturbed arterial radius and $\varepsilon$ measures its relative variations. The temporal variation of the arterial radius, described in our model by the order-unity normalized function $h ( t )$, has been found to exhibit a similar waveform at various locations^9,58–60^. Our analysis employs the waveform shown in Fig. 3a, which was adapted by Bilston et al.^35^ from the work of Millasseau et al.^61^.

The determination of the offset between arterial pulsations and transmantle pressure fluctuations requires knowledge of the relative phase shift between $h ( t )$ and $\Pi ( t )$. As discussed above, the latter temporal function was evaluated in four subjects using PC-MRI measurements of aqueductal flow rate $Q_{3{-}4}$ (Fig. 1e), which were synchronized with the heart beat. For each of the four subjects, the imaging session included also PC-MRI measurements of blood flow rate $Q_A ( t )$ entering the brain at the C2 level (see the following section for details of measurement procedure). The resulting temporal functions, also synchronized with the heart beat, are shown in Fig. 1d. In relating $Q_A ( t )$ with $h(t)$ we note that blood flow entering the brain is associated with an increase in arterial pressure, with the onset of systolic flow rate occurring simultaneously with that of the systolic pressure wave^62–65^, the latter causing the enlargement of the cerebral arteries^66,67^. Since the arterial wave speed $c_e$ is a few meters per second, with typical values lying in the range $c_e \sim 5{-}10$ m/s in healthy young adults^68^, the time lag between the pressure pulse at the C2 level and that inside the cerebral arterial tree can be anticipated to be at most on the order of a few tens of microseconds, so that the initiation of the systolic pulse of blood flow at C2 happens almost exactly when the arterial radius reaches its minimum. If the normalized time lag $\phi$ (the time lag scaled with the cardiac period) is taken to be zero in the first approximation, then one may synchronize $h(t)$ with $Q_A ( t )$ by aligning the instants of time where both signals reach a minimum, leading to the curve depicted in dark red in Fig. 3a. The influence of the time lag associated with the finite arterial wave speed, larger at larger distances from the C2 level, can be incorporated in the analysis by introducing a small shift $\phi$ in the $h(t)$ waveform, as indicated in the light-red curve of Fig. 3a. Since the functions $Q_{3{-}4} ( t )$ and $Q_A ( t )$, shown in Fig. 1e and d, are both synchronized with the heart beat, we conclude that the functions $h(t)$ and $\Pi ( t )$, shown in Fig. 3a (solid curve) and Fig. 3b, are also synchronized and exhibit the correct phase shift.

### MRI measurements

The acquisition of the flow rates $Q_{3{-}4} ( t )$ and $Q_A ( t )$ used in the analysis began by the selection of four subjects (2 women, 2 men; age range 23–38 years; weight range 52–80 kg; and height range 160–177 cm) with good health, normal pulse and respiratory rates, no cerebrospinal disorders, and no contraindications to MR imaging. The subjects were enrolled for MR imaging data collection at the Huntington Medical Research Institutes in Pasadena. The protocol and consent were approved by Quorum Review (now Advarra) Institutional Review Board. Written informed consent was obtained from all participants before MR imaging. All experiments were performed in accordance with relevant guidelines and regulations. The MR images obtained in the subjects were reviewed by a neuroradiologist to exclude cerebrospinalabnormalities.

Imaging was performed on a 3 T Signa scanner (software Version HD23; GE Healthcare) with an 8-channel head coil. CSF velocity data were acquired at the cerebral aqueduct and blood velocity at the C2 spinal level applying a cardiac-gated 2D phase-contrast MR imaging sequence with a slice orientation perpendicular to the long axis of the aqueduct / spinal canal. The imaging protocol consisted of a 2D single-shot gradient recalled-echo sequence with a slice thickness of $5 \text { mm}$ and an in-plane resolution of $0.3906 \text { mm} \times 0.3906 \text { mm}$ (aqueduct) and $0.7031 \text { mm} \times 0.7031 \text { mm}$ (C2 level). The specific imaging parameters include: $\text {TR}/\text {TE} = 32/7.5 \text { ms}$ (aqueduct) and $\text {TR}/\text {TE} = 32/6.5 \text { ms}$ (C2 level), $\text {flip angle} = 20^\circ$, $\text {velocity encoding} = 15 \text { cm}/\text {s}$ (aqueduct) and $\text {velocity encoding} = 80 \text { cm}/\text {s}$ (C2 level). Peripheral pulse gating with 30 retrospective cardiac phases ensured synchronization of CSF and blood velocity measurements with the cardiac cycle. The heart rate of the subjects during the study varied between 58 and 78 bpm.

The MR phase and magnitude data were processed with in-house MATLAB code^69,70^ to obtain the 2D distribution of longitudinal CSF velocity through the aqueduct, and the distribution of blood velocity through the internal carotid arteries and vertebral arteries at the C2 spinal level. These velocity distributions were then numerically integrated to obtain the flow rate $Q_{3{-}4} ( t )$ through the aqueduct, and the total blood flow $Q_A ( t )$ entering the cranial vault (as the sum of the flow in the two internal and two vertebral arteries), over the course of one cardiac cycle, giving the results shown in Fig. 1c,e.

### Bulk motion in periarterial spaces

#### Flow in a single periarterial element

In assessing the net flow in the PVS of Fig. 2a, we begin by focusing on a single PVS element stretching between consecutive arterial bifurcations. For simplicity, the description assumes that the PVS element is an axisymmetric annulus, as shown in Fig. 2b. The subscript $n$ will be used to denote the position of the element within the network, so that $n=1$ represents the primary branch originating at the cerebral SAS, $n=2$ is used for the secondary branches emerging after the first arterial bifurcation and so on. Correspondingly, the length, external radius, and time-dependent inner radius of branch $n$ are denoted by $\ell _n$, $r_{e,n}$, and $r_{i,n}=r_{o,n} [1+\varepsilon h( t ) ]$, respectively. Note that the model accounts for the decrease of the mean arterial radius between consecutive elements along the bifurcating tree (i.e. $r_{o,n}>r_{o,n+1}$), but assumes for simplicity a uniform relative deformation $\varepsilon$.

To derive an expression for the bulk flow of CSF in the periarterial element as a function of the pressure drop across the element $\delta p_n ( t )$ and the arterial deformation $h(t)$, we use cylindrical coordinates $(x, r)$, as indicated in Fig. 2b, with corresponding velocity components $(u, v)$ and pressure $9$. We begin by integrating the continuity equation

6 $$\begin{aligned} \frac{\partial }{\partial x}(ru)+\frac{\partial }{\partial r}(rv)=0 \end{aligned}$$

across the PVS, subject to the conditions $v={\textrm{d}} r_{i,n} /{\textrm{d}} t$ at $r=r_{i,n}$ and $v = 0$ at $r = r_{e,n}$ to yield

7 $$\begin{aligned} \frac{\partial }{\partial x} \left( \int _{r_{i,n}}^{r_{e,n}} u \, r {\textrm{d}}r \right) -r_{i,n} \frac{{\textrm{d}} r_{i,n}}{{\textrm{d}} t}=0. \end{aligned}$$

Substituting $r_{i,n}=r_{o,n} [1+\varepsilon h ( t ) ]$ and integrating the resulting equation from $x=0$ gives

8 $$\begin{aligned} Q_{n}(x,t) = C_n(t) + 2\pi \varepsilon r_{o,n}^2\left( 1+\varepsilon h\right) \frac{\text {d}h}{\text {d}t}x \end{aligned}$$

for the volumetric flow rate

9 $$\begin{aligned} Q_n ( x,t ) = \int _{r_{i,n}}^{r_{e,n}} 2 \pi \, u \, r \, {\textrm{d}}r, \end{aligned}$$

where $C_n( t ) =Q_n( 0, t )$ is the instantaneous value of $Q_n$ at $x=0$, which needs to be determined.

The analysis continues by consideration of the momentum balance equation. We focus directly on the conditions encountered in periarterial CSF flow, including viscous times $(r_{e,n}-r_{o,n})^{2}/\nu$ that are much smaller than the characteristic oscillation time $\omega ^{-1}$, so that the motion is dominated by viscous forces with negligible effects of flow acceleration. In addition, the flow is slender, in that the typical lengths $\ell _n$ are much larger than the characteristic transverse distance $r_{e,n}-r_{o,n}$, with the consequence that transverse pressure differences can be neglected (i.e. the pressure $p(x, t)$ is only a function of $x$ and $t$ in the first approximation), along with the contribution of longitudinal derivatives to the viscous stresses, thereby yielding the simplified equation

10 $$\begin{aligned} \dfrac{1}{r}\dfrac{\partial }{\partial r}\left( r\dfrac{\partial u}{\partial r}\right) = \frac{1}{\mu }\dfrac{\partial p}{\partial x}. \end{aligned}$$

Straightforward integration in the radial direction with the nonslip condition $u=0$ at $r=r_{i,n}$ and $r=r_{e,n}$ provides

11 $$\begin{aligned} u ( x,r,t ) =\frac{1}{4\mu }\left[ r^{2}-r_{e,n}^{2}\frac{\log \left( r/r_{i,n}\right) }{\log \left( r_{e,n}/r_{i,n}\right) }-r_{i,n}^{2}\frac{\log \left( r_{e,n}/r\right) }{\log \left( r_{e,n}/r_{i,n}\right) }\right] \frac{\partial p}{\partial x}, \end{aligned}$$

which can be used in Eq. (9) to give

12 $$\begin{aligned} Q_n ( x,t ) =-\frac{1}{{\mathscr {R}}_n}\frac{\partial p}{\partial x},\quad \text {with}\quad {\mathscr {R}}_n ( t ) =\frac{8\mu }{\pi }\left[ r_{e,n}^{4}-r_{i,n}^{4}-\frac{\left( r_{e,n}^{2}-r_{i,n}^{2}\right) ^{2}}{\log \left( r_{e,n}/r_{i,n}\right) }\right] ^{-1} \end{aligned}$$

denoting the hydraulic resistance of the PVS branch, which varies in time through $r_{i,n}=r_{o,n} [1+\varepsilon h ( t ) ]$.

Substitution of Eq. (12) into Eq. (8) provides an equation relating the longitudinal pressure gradient with the unknown entrance flow rate $C_n$. Integrating between $x=0$ and $x=\ell _n$ with the condition $\delta p_n=p(0,t)-p(\ell _n,t)$ yields

13 $$\begin{aligned} C_n ( t ) =-\frac{1}{\ell _n}\frac{\delta p_n}{{\mathscr {R}}_n}-\pi \varepsilon \ell _n r_{o,n}^2\left( 1+\varepsilon h\right) \frac{\text {d}h}{\text {d}t}, \end{aligned}$$

whose substitution into Eq. (8) finally completes the determination of the periarterial flow rate

14 $$\begin{aligned} Q_n ( x,t ) =-\frac{1}{\ell _n}\frac{\delta p_n}{{\mathscr {R}}_n}+2\pi \varepsilon \ell _n r_{o,n}^2\left( \frac{x}{\ell _n}-\frac{1}{2}\right) \left( 1+\varepsilon h\right) \frac{\text {d}h}{\text {d}t}. \end{aligned}$$

As can be seen, the above expression features contributions arising from the time-dependent pressure loss $\delta p_n$ as well as from the temporal variation of the arterial wall displacement $h(t)$, the latter appearing directly in the second term and indirectly in the first term, through the dependence of ${\mathscr {R}}_n$ on $r_{i,n}$. The second term in Eq. (14) has a vanishing cycle-averaged value, because $h(t)$ is a periodic function of time, while the first term exhibits in general a uniform, non-zero cycled-averaged value

15 $$\begin{aligned} \left\langle Q\right\rangle _n =-\frac{1}{\ell _n}\left\langle \frac{\delta p_n}{{\mathscr {R}}_n}\right\rangle , \end{aligned}$$

a consequence of the interplay of the arterial pulsations and the pressure fluctuations.

#### Flow in the multi-branch network

Rather than a single branch, a PVS consists of multiple elements connected by bifurcating junctions, as shown in Fig. 2a. In our model, each path from the SAS to the brain interior encounters $N$ bifurcations, so that the flow along each penetrating perivascular path crosses $N+1$ PVS elements. While it is known that, in reality, each parent vessel bifurcates into branches of different diameters^71^, for simplicity in deriving a closed-form solution, we consider the canonical case where all elements within each bifurcation level $n\in \left[ 1,N+1\right]$ are identical, ensuring that the flow rate is distributed equally between the bifurcating branches, i.e. $Q_{n+1} ( 0,t ) =Q_{n} ( \ell _{n},t ) /2$. This implies that the net flow rate through a given branch $\langle Q_n \rangle$ is related to the total net flow rate $\langle Q \rangle =\langle Q_1 ( 0,t ) \rangle$ by

16 $$\begin{aligned} \langle Q_n \rangle =\frac{\langle Q \rangle }{2^{n-1}}. \end{aligned}$$

In relating $\langle Q \rangle$ with the transmantle pressure $p_{\text {V}}-p_{\text {SAS}}$ we further assume that all branches of the network have the same length $\ell =L/\left( N+1\right)$ and that their radii ratio $r_{e,n}/r_{o,n}$ takes a constant value $r_{e}/r_{o}$, the latter assumption allowing us to write the Eq. (12) for the hydraulic resistance in the form

17 $$\begin{aligned} {\mathscr {R}}_{n}\left( t\right) =\left( \frac{\mu }{r_{o,n}^4} \right) {\mathscr {R}}^{*}, \end{aligned}$$

where

18 $$\begin{aligned} {\mathscr {R}}^{*}( t )=\frac{8}{\pi }\left[ \left( r_{e}/r_{o}\right) ^{4}-\left( 1+\varepsilon h\right) ^{4}-\frac{\left[ \left( r_{e}/r_{o}\right) ^{2}-\left( 1+\varepsilon h\right) ^{2}\right] ^{2}}{\log \left[ \left( r_{e}/r_{o}\right) /\left( 1+\varepsilon h\right) \right] }\right] ^{-1} \end{aligned}$$

is a dimensionless resistance factor, identical for all branches. Its value is highly sensitive to changes in $r_e/r_o,$ as illustrated in Fig. 2d, which shows the variation of the cycle-averaged dimensionless resistance $\langle {\mathscr {R}}^{*} \rangle$ with $r_e/r_o$ for $\varepsilon =0.1$.

The analysis continues by using Eq. (17) and $\ell =L/\left( N+1\right)$ to write Eq. (15) in the form

19 $$\begin{aligned} \left\langle Q\right\rangle _n =-\frac{r_{o,n}^4}{\mu } \left( \frac{N+1}{L}\right) \left\langle \frac{\delta p_n}{{\mathscr {R}}^{*}}\right\rangle . \end{aligned}$$

The expected reduction in arterial radius $r_{o,n}$ at each bifurcation can be described by introducing the ratio $k=r_{o,n}/r_{o,n+1}>1$, assumed to be constant for simplicity, so that we can write

20 $$\begin{aligned} r_{o,n}=\frac{r_o}{k^{n-1}} \end{aligned}$$

in terms of the radius of the initial penetrating artery $r_o=r_{o,1}$. Substituting Eqs. (16) and (20) into Eq. (19) and rearranging the result gives

21 $$\begin{aligned} \left\langle \frac{\delta p_n}{{\mathscr {R}}^{*}}\right\rangle =-\left( \frac{k^{4}}{2}\right) ^{\left( n-1\right) } \left( \frac{\mu L \left\langle Q\right\rangle }{(N+1)r_o^4}\right) . \end{aligned}$$

Since

22 $$\begin{aligned} \sum _{n=1}^{N+1}\delta p_{n}=p_{\text {V}}-p_{\text {SAS}}, \end{aligned}$$

summation of (21) over all branching levels yields

23 $$\begin{aligned} \left\langle \frac{p_{\text {V}}-p_{\text {SAS}}}{{\mathscr {R}}^{*}}\right\rangle =-\frac{\mu L \left\langle Q\right\rangle }{{\mathscr {K}} r_o^4}, \end{aligned}$$

involving the constant

24 $$\begin{aligned} {\mathscr {K}}=\frac{N+1}{\sum _{n=1}^{N+1}\left( {k^{4}}/{2}\right) ^{\left( n-1\right) }}=\frac{\left( N+1\right) \left( 2-k^{4}\right) }{2-2^{-N}k^{4\left( N+1\right) }}, \end{aligned}$$

a function of $k$ and $N$ that reduces to ${\mathscr {K}}=1$ for a single-element configuration (i.e. $N=0$). Finally, substituting into Eq. (23) the transmantle pressure $p_{\text {V}}-p_{\text {SAS}} = \Delta p_{s} + \Pi ( t )$ and solving for $\left\langle Q\right\rangle$ provides the desired expression

25 $$\begin{aligned} \left\langle Q\right\rangle =-\frac{{\mathscr {K}}r_o^4}{\mu L}\left[ \frac{\Delta p_{s}}{\left\langle {\mathscr {R}}^{*}\right\rangle }+\left\langle \frac{\Pi }{{\mathscr {R}}^{*}}\right\rangle \right] , \end{aligned}$$

while the corresponding temporal variation of the flow rate at the entrance of the network, denoted as $Q_0(t)=C_1(t)$, can be obtained using the condition (22) in Eq. (13) to give

26 $$\begin{aligned} Q_0( t ) =-{\mathscr {K}} \left[ \frac{r_o^4 }{\mu L{\mathscr {R}}^{*}}\left( \Delta p_{s} + \Pi \right) + \pi \varepsilon {\mathscr {C}} L r_o^2 \left( 1+\varepsilon h\right) \frac{\text {d}h}{\text {d}t}\right] , \end{aligned}$$

where we have introduced the additional constant

27 $$\begin{aligned} {\mathscr {C}}=\frac{1}{\left( N+1\right) ^{2}\left( k^{2}-2\right) }\left[ \frac{2k^{2}\left( 1-\left( k^{4}/2\right) ^{N+1}\right) }{1-k^{4}/2}-\frac{\left( 1-k^{2\left( N+1\right) }\right) \left( k^{2}+2\right) }{1-k^{2}}\right] . \end{aligned}$$

Equation (25) can be conveniently written in the alternative form

28 $$\begin{aligned} \left\langle Q\right\rangle =\frac{{\mathscr {K}}r_o^4}{\mu L \left\langle {\mathscr {R}}^{*}\right\rangle }\left( \Delta p_{p}-\Delta p_{s}\right) , \end{aligned}$$

where

29 $$\begin{aligned} \Delta p_{p}=-\left\langle {\mathscr {R}}^{*}\right\rangle \left\langle \frac{\Pi }{{\mathscr {R}}^{*}}\right\rangle \end{aligned}$$

represents an apparent pressure difference generated as a result of the cooperative synchronization of the arterial pulsations and transmantle-pressure fluctuations. Positive values of $\left\langle Q\right\rangle$ correspond to bulk motion towards the interior of the brain, while negative values suggest outflow towards the SAS. As expected, since $\Delta p_s>0$, the small positive average pressure existing in the ventricles tends to produce outflow towards the SAS (i.e. negative values of $\left\langle Q\right\rangle$). Hence, bulk periarterial motion towards the interior of the brain (i.e. positive values of $\left\langle Q\right\rangle$) may occur only when the pressure difference induced by dynamic pumping $\Delta p_{p}$ is positive and larger than $\Delta p_{s}$.

#### A lumped-parameter model for pericapillary and transparenchymal flow

The above analysis employs the transmantle pressure-defined as the pressure difference between the lateral ventricles and the SAS-as a proxy for the pressure difference across the periarterial network. This approach, utilized in other studies^13,14^, does not account for the fact that the periarterial spaces are not directly connected to the ventricles. Instead, perivascular spaces surrounding arteries connect to those around arterioles, which in turn transition into pericapillary spaces. As arterioles give way to capillaries, vessel pulsatility gradually diminishes, with capillaries exhibiting little to no pulsatile motion. Consequently, pericapillary spaces do not contribute to the cooperative pumping mechanism described above; rather, they introduce significant resistance to flow within the perivascular network.

Additionally, a portion of CSF circulating in periarterial spaces permeates the brain parenchyma via aquaporin-4 (AQP4) channels on astrocyte endfeet, acting as distributed sources that drive CSF flow through the interstitial space of brain tissue. These alternative pathways introduce additional pressure losses that are not accounted for when assuming a direct connection between the periarterial spaces and the ventricles.

The effects of pericapillary and transparenchymal flow onperiarterial pumping can be incorporated in an approximate way by including a constant downstream resistance $R_{\textrm{d}}$ at the downstream end of the periarterial network, as shown schematically in the inset of Fig. 5. This addition causes the pressure at the distal end of the periarterial network, $p_{L}$, to differ from that found in the ventricles, $p_{\text {V}}$, so that the condition (22) must be replaced with

30 $$\begin{aligned} \sum _{n=1}^{N+1}\delta p_{n} = p_{L} - p_{\text {SAS}} = \left( p_{\text {V}} - p_{\text {SAS}}\right) - \left( p_{\text {V}} - p_{L}\right) = \Delta p_{s} + \Pi + R_{\text {d}} Q_L. \end{aligned}$$

As is consistent with the negligible inertia of pericapillary and transparenchymal flows, in writing (30) the linear relation

31 $$\begin{aligned} p_{\text {V}} - p_{L} = -R_{\text {d}} Q_L \end{aligned}$$

is assumed between the instantaneous values of the downstream pressure drop $p_{\text {V}} - p_{L}$ and the flow rate at the distal end of the periarterial network $Q_L ( t ) =2^N Q_{N+1} (\ell ,t )$.

To determine the bulk flow rate, we begin by noting that the flow rate at the entrance of the $n$-th level branch, $Q_n ( 0, t )$, is related to the total entrance flow rate $Q_0$ by

32 $$\begin{aligned} Q_{n} ( 0,t ) = \frac{Q_{0}}{2^{n-1}} + \pi \varepsilon \ell \left( 1+\varepsilon h\right) \frac{\text {d}h}{\text {d}t} \frac{1}{2^{n-1}} \sum _{m=1}^{n-1} 2^{m} r_{o,m}^{2}, \end{aligned}$$

while the distal flow rate, needed to compute the downstream pressure loss (31), is given by

33 $$\begin{aligned} Q_{L} ( t ) =Q_{0}+\pi \varepsilon \ell \left( 1+\varepsilon h\right) \frac{\text {d}h}{\text {d}t}\left[ \sum _{m=1}^{N+1}2^{m} r_{o,m}^{2}\right] . \end{aligned}$$

These relations follow from the equal distribution of flow between bifurcating branches, i.e. $Q_{n+1} ( 0,t ) = Q_{n} ( \ell ,t ) /2$, along with the relation

34 $$\begin{aligned} Q_{n} ( \ell ,t ) - Q_{n} ( 0,t ) = 2\pi \varepsilon \ell r_{o,n}^{2} \left( 1+\varepsilon h\right) \frac{\text {d}h}{\text {d}t} \end{aligned}$$

describing the flow rate change along each branch segment, the latter obtained by subtracting (14) evaluated at $x=0$ from (14) evaluated at $x=\ell$.

Substituting (32) back into Eq. (14) yields an expression for the $n$-th level pressure jump $\delta p_n$ in terms of $Q_{0}$ and the geometric parameters. Adding the $\delta p_n$ contributions across all levels and applying (30) leads to an expression for $Q_{0}$, which simplifies to

35 $$\begin{aligned} Q_{0} ( t ) =-{\mathscr {K}}\left[ \frac{r_{o}^{4}}{\mu L}\left( \frac{\Delta p_{s}+\Pi }{{\mathscr {R}}^{*}}\right) +\pi \varepsilon Lr_{o}^{2}\left( 1+\varepsilon h\right) \frac{\text {d}h}{\text {d}t}\left( {\mathscr {C}}+\frac{R_{\text {d}}^{*}}{{\mathscr {R}}^{*}}\tilde{{\mathscr {C}}}\right) \right] /\left( 1+\frac{R_{\text {d}}^{*}}{{\mathscr {R}}^{*}}{\mathscr {K}}\right) \end{aligned}$$

under the modeling assumptions

36 $$\begin{aligned} \ell =L/\left( N+1\right) ,\quad r_{o,n}/r_{o,n+1}=k,\quad \text {and}\quad r_{e,n}/r_{o,n}=r_{e}/r_{o}. \end{aligned}$$

Above, we have introduced the dimensionless downstream resistance

37 $$\begin{aligned} R_{\textrm{d}}^{*} = \frac{R_{\text {d}} r_{o}^{4}}{\mu L} \end{aligned}$$

along with the constant

38 $$\begin{aligned} \tilde{{\mathscr {C}}}=\frac{2k^{2}\left( 1-\left( 2/k^{2}\right) ^{N+1}\right) }{(N+1)\left( k^{2}-2\right) }. \end{aligned}$$

Finally, the bulk flow rate can be obtained from by averaging on time (35), yielding

39 $$\begin{aligned} \left\langle Q\right\rangle =-{\mathscr {K}}\left\langle \left[ \frac{r_{o}^{4}}{L\mu }\left( \frac{\Delta p_{s}+\Pi }{{\mathscr {R}}^{*}}\right) +\pi \varepsilon Lr_{o}^{2}\left( 1+\varepsilon h\right) \frac{\text {d}h}{\text {d}t}\left( {\mathscr {C}}+\frac{R_{\text {d}}^{*}}{{\mathscr {R}}^{*}}\tilde{{\mathscr {C}}}\right) \right] /\left( 1+\frac{R_{\text {d}}^{*}}{{\mathscr {R}}^{*}}{\mathscr {K}}\right) \right\rangle , \end{aligned}$$

which naturally reduces to (25) when $R_{\textrm{d}}^*=0$.

The evaluation of (39) requires an estimate for the resistance $R_{\text {d}}^{*}$. While estimating the pressure losses associated with interstitial flow is not straightforward, the additional resistance introduced by pericapillary flow can be determined by extending our periarterial network model to include capillary perivascular spaces. Maintaining the structural characteristics and total length $L$ of the periarterial network, one can incorporate pericapillary spaces by adding $M$ additional bifurcating levels, where each branch symmetrically divides into two daughter branches, consistent with the previous arteriolar levels. The key distinction is that, unlike arteriolar branches, the new capillary branches do not exhibit pulsatility ($\varepsilon =0$), resulting in a hydraulic resistance that remains constant over time. If the relations in (36) are assumed to hold also for the capillary levels $n \in [N+2, N+M+1]$, then following the derivation that led to (25), one can show that the bulk flow rate corresponding to the extended perivascular network takes the form (39) with

40 $$\begin{aligned} R_{\text {d}}^{*} = \frac{\left( k^{4}/2\right) ^{N+1}\left( 1-\left( k^{4}/2\right) ^{M}\right) }{\left( N+1\right) \left( 1-k^{4}/2\right) }\frac{8}{\pi }\left[ \left( r_{e}/r_{o}\right) ^{4}-1-\frac{\left[ \left( r_{e}/r_{o}\right) ^{2}-1\right] ^{2}}{\log \left( r_{e}/r_{o}\right) }\right] ^{-1} \end{aligned}$$

representing in this case the additional dimensionless resistance introduced by non-pulsatile capillaries.

## Results

### Selection of physiological parameters

The value of $\left\langle Q\right\rangle$ is influenced by severalphysiological parameters, which must be chosen from the limited available data. Specifically, the expression (25) exhibits explicit dependences on the arterial path length $L$ and on the radius of the primary penetrating artery $r_o$. In addition, the solution depends on the external-to-inner wall-radius ratio in the annular PVS $r_e/r_o$ and the relative amplitude of the arterial deformations $\varepsilon$, which are incorporated through the dimensionless resistance ${\mathscr {R}}^{*}$ as defined in Eq. (18). The characteristics of the PVS network enter in Eq. (25) through the factor ${\mathscr {K}}$, which is governed by two additional physiological parameters, namely, the radii ratio $k = r_{o,n}/r_{o,n+1}$ between consecutive arterial branches and the number of bifurcations $N$ along each path connecting the SAS to the brain’s interior.

In the following evaluations of Eq. (25) we assume $L = 5$ cm, which corresponds to the characteristic distance from the lateral ventricles to the cortical surface^72^. To guide the selection of $r_o$, $r_e/r_o$, and $\varepsilon$, Table 1 provides physiologically relevant values for these three parameters at specific vascular locations. As some of these values are not reported explicitly in the references, we briefly explain how they were obtained. For Bedussi et al.^73^ in humans, we used the linear relationship between PVS width ($r_e - r_o$) and vessel diameter ($2r_o$) shown in the Supplementary Figure 1B^73^, approximated by $r_e - r_o = 0.77 \cdot (2r_o) - 0.054$ mm, which gives $1.46< r_e/r_o < 2.32$ for the range of vessel diameters 100 $\upmu$m $< 2r_o <$ 500 $\upmu$m reported. For Mestre et al.^9^, a PVS-to-artery area ratio of 1.4 corresponds to $r_e/r_o = 1.55$ under the assumption of a concentric circular annulus. Similarly, the average area ratio of 1.26 reported by Schain et al.^74^ yields $r_e/r_o = 1.50$, while the 95% confidence interval area ratio of 0.973 to 1.12 reported by Raicevic et al.^75^ corresponds to $1.40< r_e/r_o < 1.46.$ In the following evaluations we use $r_o = 200$ $\upmu$m, $r_e/r_o = 2$, and $\varepsilon = 0.1$, the last value suggested by Bilston et al.^76^ as representative of small arteries within the human vasculature, which is the focus here.

The parameter $k$ is derived from Murray’s Law^77^, $r_{0}^{3} = r_{1}^{3} + r_{2}^{3}$, which defines the relationship between the radius of a parent vessel ($r_{0}$) and those of its branches ($r_{1}$ and $r_{2}$). This principle, validated by Rossitti et al.^78^ for the internal carotid artery system, results in $k = 1.26$ under our model assumption $r_{1} = r_{2}$. Estimating the number of bifurcations $N$ along the PVS network requires further consideration. While studies have examined the overall number of bifurcations in the human cerebral arterial tree^71,79^, specific data on the bifurcation count within individual penetrating arteries are still limited. To bridge this gap, we note that the number of bifurcations determines the radius of the terminal branches in the network $r_{o,N+1}=r_o/k^N$, so that the value of $N$ can be selected to match the typical radius of the smallest arterioles (i.e. 15 $\upmu$m^80^). For $r_o = 200$ $\upmu$m and $k = 1.26$, this approach results in $N = 11$, for which $r_{o,N+1} = 15.7$ $\upmu$m. For this number of bifurcations, the average branch length $L/\left( N+1\right) = 4.2$ mm aligns well with values observed in cerebral artery branching^71^, thereby providing additional confidence in the selection.

**Table 1:** Physiological parameters of the periarterial spaces reported in the literature for different species and locations in the vascular system, including information on the artery radius $r_o$, the ratio $r_e/r_o$, and the amplitude of arterial pulsations $\varepsilon$.

Species	Reference	Location	$r_{o}$ [$\upmu$m]	$r_{e}/r_{o}$	$\varepsilon$
Humans	Thorin et al.^81^	Pial arteries	200–1200	–	–
	Miao et al.^80^	Semioval center	15–150	–	–
	Bedussi et al.^73^	Leptomeningeal vessels	50–250	1.46–2.32	–
Rats	Lam et al.^82^	Spinal cord	2–20	1.10	–
Mice	Bedussi et al.^73^	Leptomeningeal vessels	10–20	–	0–0.1
	Mestre et al.^9^	Middle cerebral artery	20	1.55	0.02
	Schain et al.^74^	Pial arteries	6–11	1.50	–
	Raicevic et al.^75^	Pial arteries	10	1.40–1.46	–

### Assessment of bulk flow in periarterial spaces

#### Results for four healthy subjects

As previously explained, the functions $h(t)$ and $\Pi ( t )$ shown in Fig. 3a (solid curve) and 3b are based on simultaneous PC-MRI measurements of arterial blood flow (Fig. 1d) and aqueductal CSF flow (Fig. 1e) in four healthy volunteers. The use of these curves, together with the parametric values $r_o = 200$ $\upmu$m, $r_e/r_o = 2,$ $\varepsilon = 0.1$, $L = 5$ cm, $k = 1.26$, and $N = 11$, yields the results shown in Fig. 3c. The curves shown on the left-hand-side plot represent the evolution with time of the flow rate at the entrance of the network $Q (0, t)$ evaluated from Eq. (26). As can be seen, peak-to-peak amplitudes are in the range of tens of microliters per minute. The associated cycle-averaged flow rate $\langle Q \rangle$ is much smaller, as can be seen by evaluating Eq. (25) to give the values listed in the table of Fig. 3c. The table also shows the corresponding values of the apparent pressure difference $\Delta p_p$ induced by the interplay of the arterial pulsations and transmantle-pressure fluctuations, which can be evaluated from Eqs. (29) and (18) for given values of $h(t)$, $\Pi ( t )$, $\varepsilon$ and $r_e/r_o$. For all subjects, the resulting values are larger than the estimated value of the mean transmantle pressure $\Delta p_s=0.015$ Pa, thereby yielding positive values of $\langle Q \rangle$, as dictated by Eq. (28). In all cases, therefore, bulk motion is directed towards the interior of the brain, as shown schematically in the right-hand-side PVS representation of Fig. 3c. This suggests that the dynamic pumping resulting from the cooperative synchronization between arterial pulsations and transmantle pressure fluctuations is capable of counteracting the cycle-averaged adverse transmantle pressure, thereby providing a plausible explanation for glymphatic periarterial flow.

#### Influence of arterial pulsation delay

The phase of the solid curve $h(t)$ shown in Fig. 3a, used in the evaluations presented in Fig. 3c, assumes that the systolic pulse of blood flow occurs simultaneously everywhere in the cerebral arterial system, as determined by the blood flow-rate measurements at the C2 level. Since the arterial wave speed $c_e$ is finite, small phase lags scaling with the distance along the arterial branch $d$ can be expected to occur. For example, using $d = 20$ cm as characteristic distance together with $c_e=5$ m/s yields a phase lag $d/c_e \sim 40 \, \mu \textrm{s} \ll T$, resulting in a small shift $\phi \sim 0.04$ of the corresponding curve $h(t)$ (relative to $\Pi$). To investigate the sensitivity of the predictions to this phase lag, we plot in Fig. 3d the values of $\left\langle Q\right\rangle$ obtained when varying the phase between the transmantle pressure fluctuations and the arterial pulsation, as indicated by the dot-dashed curve of Fig. 3a. Although $\phi$ is expected to be small and positive, the evaluations include the entire range of possible values of the phase lag $-0.5 \le \phi \le 0.5.$ The results indicate that for the four subjects analyzed here the variation of $\left\langle Q\right\rangle$ is not very pronounced within the expected range of values 0 < $\phi$ ≲ 0.04, for which the periarterial flow remains always positive.

#### Impact of physiological parameters on pumping efficiency

**Fig. 4:**
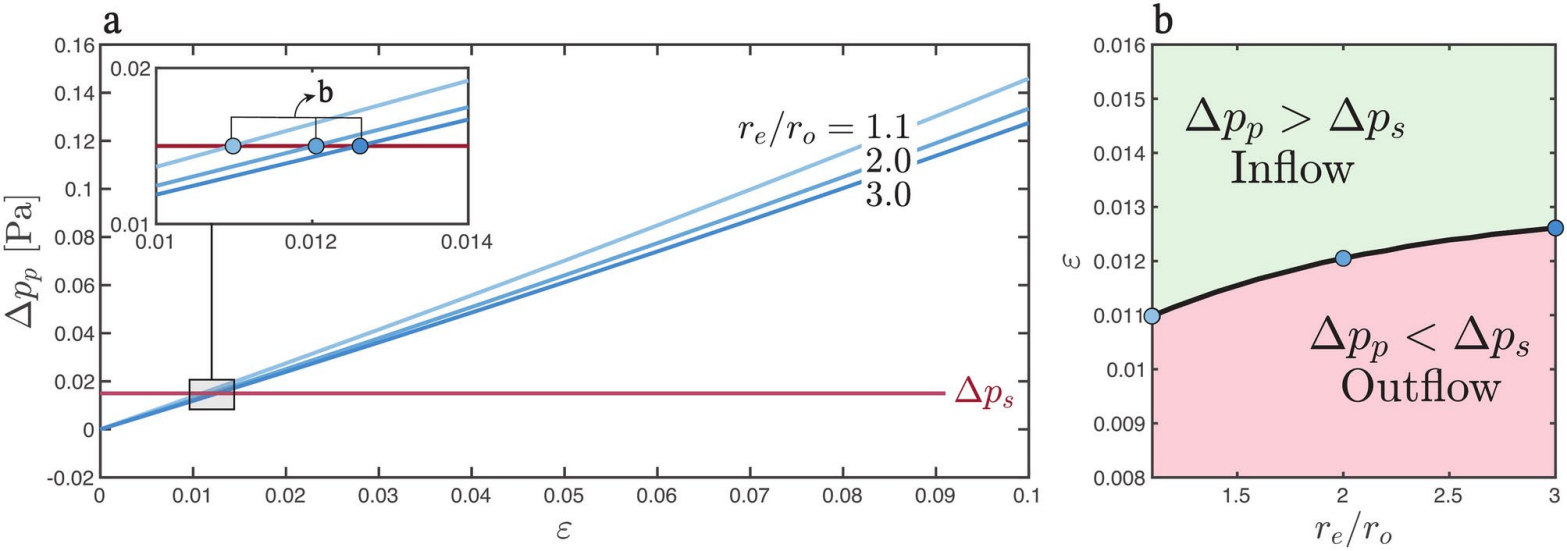
Effectiveness of the pumping mechanism within the physiologically relevant range using pressure data from subject S2. (**a**) Evolution of the apparent pressure difference, $\Delta p_p$, with varying amplitudes of arterial deformations, $\varepsilon$. The three curves, in different shades of blue, correspond to the ratios $r_e/r_o=1.1$, 2.0, and 3.0. A red horizontal line represents the mean transmantle pressure, $\Delta p_s$. (**b**) The value of $\varepsilon$, as a function of $r_e/r_o$, that results in zero bulk flow (i.e., $\Delta p_p=\Delta p_s$), with the three points corresponding to the inset in (**a**).

According to Eq. (28), the pumping mechanism analyzed here is effective only when the pressure difference induced, $\Delta p_p$, exceeds the existing mean transmantle pressure, $\Delta p_s$. As shown in Eqs. (29) and (18), for the given waveforms $h(t)$ and $\Pi ( t )$, the resulting value of $\Delta p_p$ depends only on the ratio $r_e/r_o$ and the amplitude $\varepsilon$, with the values $r_e/r_o=2$ and $\varepsilon =0.1$ selected in the computations shown in Fig. 3c,d. Different regions of the periarterial network are expected to have different values of $r_e/r_o$ and $\varepsilon$, which motivates investigating the sensitivity of the pumping efficiency to these parameters, as done in Fig. 4a using the function $\Pi$ corresponding to subject S2.

The plot reveals that the value of $\Delta p_p$, largely independent of $r_e/r_o$, increases linearly with the amplitude of the arterial deformation $\varepsilon$. There exists a minimum threshold value of $\varepsilon \simeq 0.01$ below which the pumping mechanism is not strong enough to counteract the cycle-averaged transmantle pressure (i.e. $\Delta p_p <\Delta p_s$). This threshold value varies only slightly with $r_e/r_o$, as shown in Fig. 4b.

#### Influence of multi-branch configuration on bulk flow

In the model developed above, the multiplying factor ${\mathscr {K}}$ defined in Eq. (24) carries the effect of branching on bulk flow. As shown in Eq. (28), the bulk flow rate of a multi-branch network is ${\mathscr {K}}$ times that of the corresponding single-element network. Thus, the specific network structure determines only the magnitude of bulk flow, but not its direction, which is largely governed instead by the parameters $\varepsilon$ and $r_e/r_o$ determining the apparent pressure difference $\Delta p_p$, as discussed above.

As seen in Eq. (24), the value of ${\mathscr {K}}$ is a function of the number of bifurcations in the arterial tree $N$ and the parent-to-daughter radius ratio $k$, with the values $N = 11$ and $k = 1.26$ used in the evaluations of Fig. 3c,d. The variation of ${\mathscr {K}}$ with these two parameters is investigated in Fig. 2c. Three distinct behaviors emerged for $N>0$, depending on the specific value of $k$. When $k > 2^{1/4}\simeq 1.1892$, an increased number of bifurcations induces a decrease of ${\mathscr {K}}$ (and therefore and associated decrease of bulk flow rate), whereas for $k < 2^{1/4}$, ${\mathscr {K}}$ increases with the number of bifurcations. Interestingly, for $k = 2^{1/4}$ the value ${\mathscr {K}}=1$ does not change with the number of bifurcations. For the value $k=1.26>2^{1/4}$ assumed in the above evaluations, the flow rate diminishes rapidly with the number of bifurcations, and becomes about a fifth of that found in a single-element network when $N=11$ (i.e. ${\mathscr {K}}=0.2$). These findings underscore the importance of the anatomical structure of the periarterial network.

#### Effects of pericapillary and transparenchymal flow

**Fig. 5:**
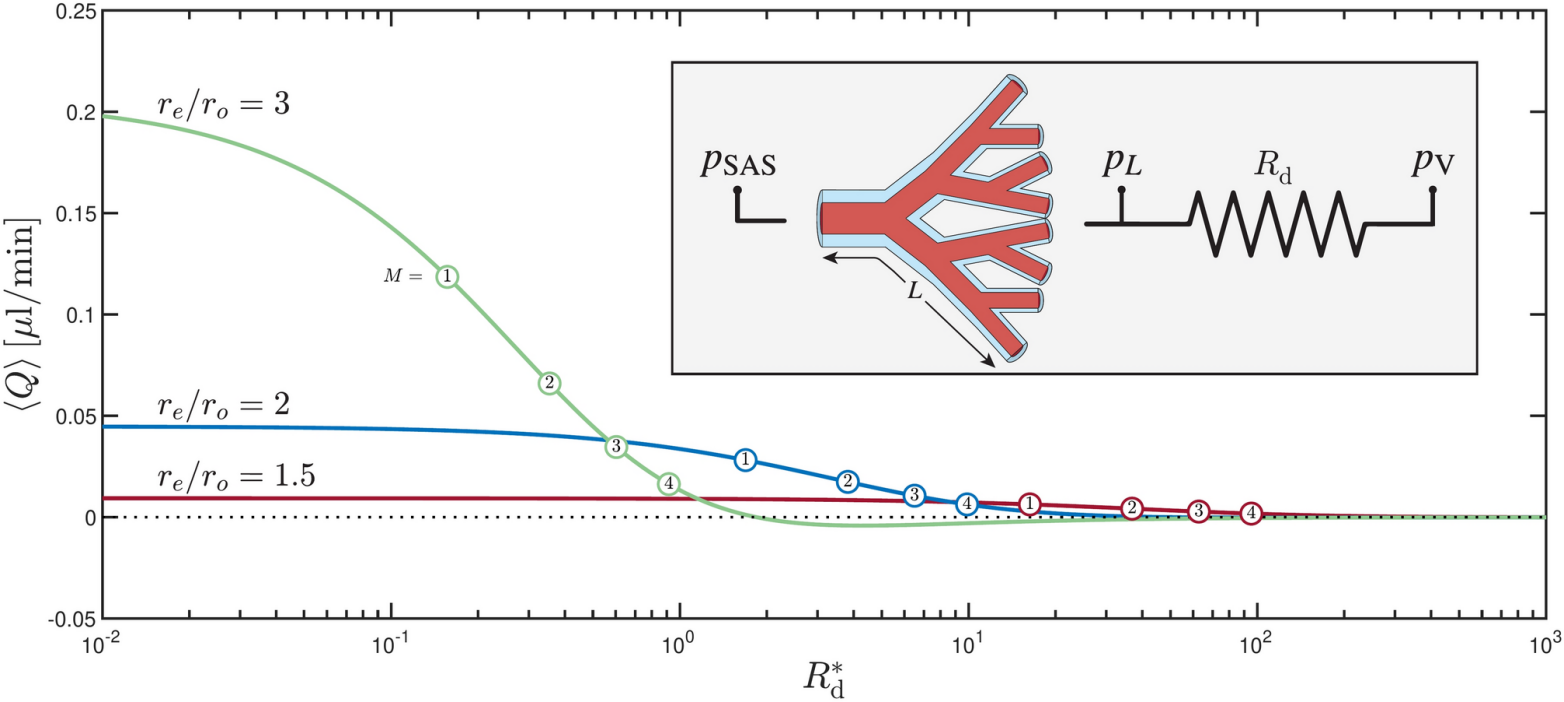
The variation of the bulk flow rate $\langle Q \rangle$ with the dimensionless downstream resistance $R_{\textrm{d}}^{*}$ as obtained from (39) for three different values of $r_e/r_o$. The analysis is based on pressure data from subject S2 using the parameters $r_{o}=200\:\upmu$m, $L=5$ cm, $\varepsilon =0.1$, $N=11$, and $k=1.26$. Circle markers indicate, for each $r_e/r_o,$ the estimated resistance $R_{\textrm{d}}^{*}$ associated with the presence of $M = 1, 2, 3, 4$ pericapillary elements at the downstream end of the perivascular network.

The results shown in Figs. 3 and 4 are evaluated using the transmantle pressure to quantify the pressure difference between the ends of the periarterial network, thereby neglecting downstream pressure losses arising from pericapillary and transparenchymal flows. As discussed earlier, these effects can be approximated by introducing a constant downstream resistance. The resulting bulk flow rate $\langle Q \rangle$, given by the closed-form expression (39), decreases as the resistance is increased, as illustrated in Fig. 5.

The curves in Fig. 5 help quantify effects of pericapillary and transparenchymal flow. For low values of $R_{\textrm{d}}^{*}$, the net flow rate approaches the value predicted by (28), yielding $\langle Q \rangle \rightarrow (0.94, 4.5, 21) \times 10^{-2}$ for $r_e/r_o = (1.5, 2, 3)$. The strong dependence of $\langle Q \rangle$ on $r_e/r_o$ is mainly a consequence of the pronounced variation of the cycle-averaged dimensionless resistance of the periarterial network $\langle {\mathscr {R}}^{*}\rangle$, shown in Fig. 2d. As $R_{\textrm{d}}^{*}$ increases the bulk flow rate steadily decreases until it eventually becomes negative, indicating that a strong downstream resistance can halt or even reverse bulk flow. Interestingly, the critical value of $R_{\textrm{d}}^{*}$ at which the net flow reverses (i.e. $\langle Q \rangle =0$) is lower as $r_e/r_o$ increases, with the critical values of $R_{\textrm{d}}^{*}\approx \left( 2.0, 0.059, 0.002\right) \times 10^3$ obtained for $r_e/r_o = (1.5, 2, 3)$.

Expected values of $R_{\textrm{d}}^{*}$ associated with the presence of nonpulsatile capillaries can be quantified using  (40), yielding the values indicated by the circle markers in Fig. 5, with $M = 1, 2, 3, 4$ denoting the number of capillary levels found at the downstream end of the perivascular network. These markers delineate the anticipated physiological range of $R_{\textrm{d}}^{*}$ and suggest that while downstream resistance may substantially diminish bulk flow, it does not necessarily invalidate the underlying mechanism, as net flow in all cases remains positive for the estimated values.

## Discussion

In this study, we developed an MRI-based model of periarterial CSF flow to determine whether the dynamic interaction between arterial pulsations and transmantle pressure fluctuations could explain centripetal glymphatic flow, counterintuitively directed against the existing cycle-averaged pressure gradient. In four healthy subjects, arterial pulsations were inferred from axial PC-MR images of cranial arterial blood flow at the C2 level. Synchronously, CSF flow was measured in the cerebral aqueduct using PC-MR, and these measurements were used to calculate transmantle pressures. Our results consistently revealed that the pumping mechanism provides a sufficient contribution to overcome the adverse mean pressure gradient, resulting in a net periarterial inflow of CSF.

Building on these results, we refined our analysis to explore potential limitations in our approach. We first examined the methodology used to calculate arterial deformations, based on PC-MRI measurements of arterial blood flow at the C2 level. While in deriving the results in Fig. 3c we assumed that the minimum arterial deformation aligns with the onset of the systolic pulse of the blood flow measurements, there is, in reality, a small phase lag, represented by the parameter $\phi$ in Fig. 3a. Specifically, because the cerebral arteries are located downstream from the blood-flow measurement site (vertebral and internal carotid arteries at the C2 level), a positive phase shift is expected. To evaluate the impact of our initial assumption of zero offset ($\phi =0$), which was utilized to derive the results in Fig. 3c, we represented in Fig. 3d periarterial bulk motion as a function of the phase lag. The results indicate that the conclusion that the dynamic interactions between the pressure gradient and the arterial pulsations provide a bulk flow towards the brain’s interior holds for the range of small positive values of $\phi \sim 0{-}0.04$ that can be expected to occur. The plots in Fig. 3d also underscore the importance of the synchronization between the pulsations of transmantle pressure and arterial radius, in that the pumping mechanism, which yields positive inflow for the four healthy patients tested here, would give negative flow (i.e., toward the cerebral SAS) should the value of $\phi$ be sufficiently large.

**Fig. 6:**
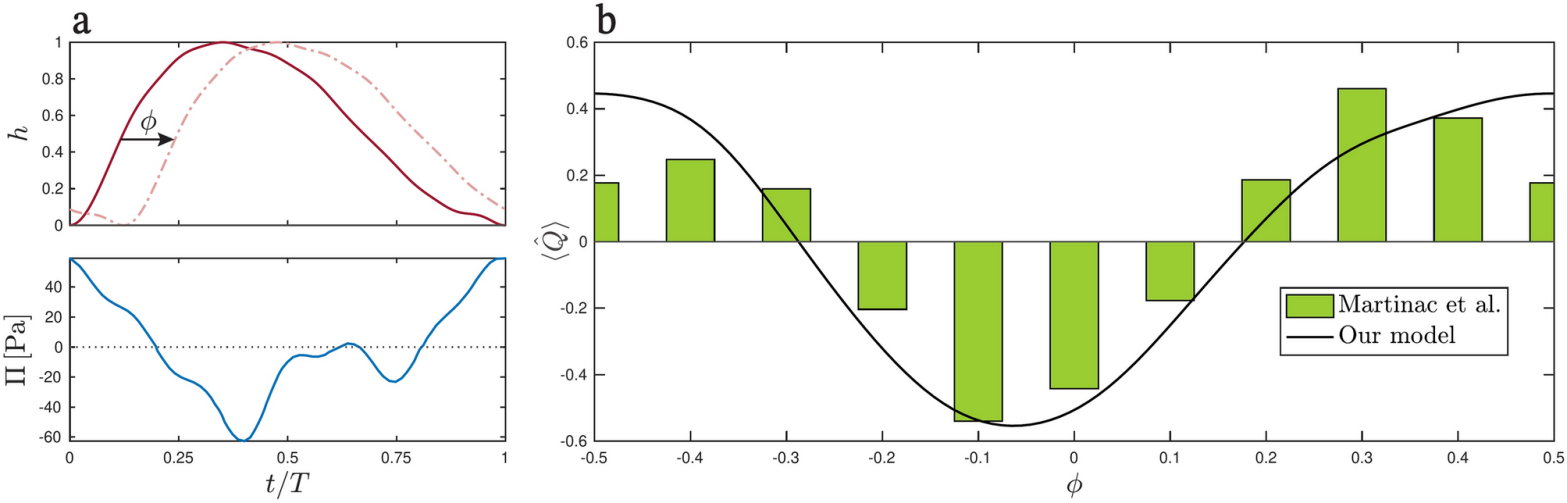
Validation of our model using data and results from Martinac et al.^39^. (**a**) Arterial pulsations and transmantle pressure used by Martinac et al., adapted from Bilston et al.^35^ and Kasprowicz et al.^40^, respectively. (**b**) Comparison of the variation with $\phi$ of the bulk flow rate (normalized to give a unity peak-to-peak amplitude) determined by Martinac et al.^39^ (green bars) with that evaluated using Eq. (25) for the waveforms shown here with $r_e/r_o = 2$, and $\varepsilon = 0.1$.

Following our initial findings, we further analyzed the relationship between the apparent pressure difference $\Delta p_p$ induced by the pumping mechanism and the parameters of the PVS. As shown in Fig. 4, within the physiologically relevant range of $r_e/r_o$ studied, the apparent pressure difference $\Delta p_p$ linearly depends on the pulsation amplitude $\varepsilon$, so that a minimum value $\varepsilon \simeq 0.01$ is needed for $\Delta p_p$ to counterbalance the overpressure $\Delta p_s \simeq 0.015$ Pa existing in the brain’s interior. The physiological measurements in Table 1 exhibit a fairly large variability in the reported values of $\varepsilon$. While some studies suggest that $\varepsilon$ could be as high as $\varepsilon = 0.1$^73,76^, Mestre et al.^9^ reports that, in mice, $\varepsilon$ is relatively small, around $\varepsilon =0.02$. This small magnitude of heartbeat-driven pulsations, which has raised discussions about the role of peristalsis in driving bulk motion^23–26^, would still be sufficient to sustain positive net flow with the pumping mechanism investigated here. However, obtaining accurate physiological parameters in humans remains essential to refine and validate these predictions.

The pumping rate is influenced by various anatomical features of the periarterial branched network, such as the size of the arterial vessels and the number and structure of bifurcations. Detailed measurements of these features in the human brain remain scarce, thereby hindering efforts to quantify precisely the induced flow. Additionally, there is uncertainty regarding the spatial extent of the periarterial network itself. Our analysis assumes that the branched periarterial network starts at the SAS at the exterior surface of the brain, in agreement with previous modeling efforts^22,23,39^. Correspondingly, in our model it is the variation of transmantle pressure, i.e. the pressure difference between the SAS and the brain’s interior, that governs flow in the periarterial network. However, recent observations by Eide et al.^43^ suggest that the periarterial network extends further upstream, along the large arterial arteries, to the basal cisterns of the brain. Their in vivo observations of tracer dispersion show tracer transport along large periarterial spaces surrounding the anterior, middle, and posterior cerebral arteries that, after subsequent bifurcation, eventually may reach the exterior surface of the brain, before penetrating the brain again in the manner postulated in our model. Future endeavors should explore the implications of these recent findings.

The precise anatomical extent of the periarterial network and its connectivity with downstream pathways also remain uncertain, raising questions about how CSF transitions from periarterial spaces to deeper brain regions. Pressure losses can be expected to occur as CSF flows along pericapillary and transparenchymal pathways. These effects were investigated by incorporating a lumped downstream resistance $R_{\textrm{d}}$ into the model, leading to a modified expression for bulk flow. The additional resistance is seen to reduce the efficiency of the dynamic pumping investigated here, although the net flow remains positive (directed inward) for estimated values of $R_{\textrm{d}}.$ The results underscore the role of pericapillary and interstitial resistance in modulating glymphatic flow and highlights the need for improved quantification of these losses.

Recent findings provide strong evidence that vasomotion—the rhythmic dilation and constriction of blood vessels driven by neural activity and mediated by vascular smooth muscle cells—plays a key role in regulating glymphatic influx along periarterial spaces. Holstein et al.^18^ showed in mice that sensory stimulation induces arterial dilation, which narrows the perivascular space and transiently reduces CSF velocity; this is then followed by arterial constriction that restores and increases CSF flow velocities, ultimately leading to a net increase in glymphatic influx. More recently, Hauglund et al.^19^ demonstrated that slow vasomotion, driven by infraslow ($\sim 0.02\,\textrm{Hz}$) oscillations in norepinephrine (NE) during NREM sleep, is critical for promoting glymphatic clearance. In their experiments, freely behaving mice exhibited NE oscillations that were tightly coupled with cyclic changes in cerebral blood volume and CSF periarterial flow, resulting in rhythmic arterial constrictions and dilations that enhance glymphatic clearance.

Although these studies underscore the importance of vasomotion in glymphatic function, the underlying physical mechanism remains open to debate. Our proposed mechanism, which operates at the cardiac frequency ($\sim 1\,\textrm{Hz}$), may be modulated by slower vasomotion ($\sim 0.02\,\textrm{Hz}$) by altering the ratio $r_e/r_o$ over successive cardiac cycles. As our results in Fig. 2d indicate, variations in $r_e/r_o$ profoundly affect flow magnitude by modifying the hydraulic resistance of the PVS. Specifically, during vasodilation a decreased $r_e/r_o$ (i.e. a reduced PVS width) leads to higher resistance and minimal bulk flow during those cardiac-cycles, whereas vasoconstriction increases $r_e/r_o$, lowering resistance and substantially boosting the magnitude of the flow. Importantly, throughout all phases the CSF flow remains directed centripetally toward the interior of the brain, as our results in Fig. 4 demonstrate, since changes in $r_e/r_o$ do not reverse the bulk flow direction. In this way, the dynamic interplay between cardiac pulsations and transmantle pressure fluctuations, coupled with a modulated PVS baseline width due to vasomotion, can drive a net inward flow-most effectively during vasoconstriction phases-in agreement with experimental observations^18,19^.

To our knowledge, the combined effect of arterial pulsations and transmural pressure fluctuations on bulk flow have been previously examined in only one study. Martinac et al.^39^ tested the pumping mechanism combining intracranial pressure measurements in a patient with hydrocephalus^40^ and the arterial pulsations of Bilston et al.^35^, both waveforms being shown in Fig. 6a. In contrast to our findings, their results suggest that the timing interplay cannot explain periarterial bulk flow, in that for small values of the phase lag $\phi$, the resulting flow rate is negative, as indicated by the green bars shown in Fig. 6b. Since the efficiency of the pumping mechanism depends critically on the relative timing between pressure and arterial pulses, as demonstrated in Fig. 3d, the conclusions of Martinac et al.^39^ can be attributed to their use of pressure data from a patient with hydrocephalus, a condition that has been related to abnormal transmantle pressure dynamics^41^ and impaired periarterial flow^43^.

Integrating Martinac’s data into our model offers a valuable opportunity to further verify Martinac’s results in connection with hydrocephalus patients and also to compare the two modelling approaches. The results are presented in Fig. 6b, where we depict bulk flow rate, normalized to give a unity peak-to-peak amplitude, as a function of the phase shift between transmantle pressure and arterial deformations, as defined in Fig. 6a. As can be seen, Martinac’s results (green bars) align closely with our predictions (continuous black curve), both approaches yielding negative values of the net flow rate for moderate values of $\phi$. The degree of agreement is remarkable given the differences between the two modeling approaches; while we rely on closed-form solutions systematically derived from the conservation equations of mass and momentum, Martinac et al. utilized direct numerical simulations in a model with three distinct regions-PVS, astrocyte endfeet, and brain parenchyma-each with different permeabilities. The agreement seems to indicate that certain simplifications, such as ignoring the permeability of the perivascular wall, do not significantly impact the solution. Overall, the strong correlation between the two approaches suggests that our canonical periarterial tree model may be sufficient to capture the overall qualitative behavior, though a more detailed approach might be needed to improve quantitative predictions.

Although our model shows promise in explaining glymphatic flow under a range of conditions, it has a number of limitations worth pointing out. The model does not address the enhanced glymphatic function observed during sleep^6–8^, and focuses primarily on cardiac-driven flow, potentially overlooking the influence of respiration on glymphatic dynamics^1,83,84^. In modeling the periarterial tree, several simplifications were made. Some, like assuming an impermeable brain parenchyma or uniform arterial wall deformations, are easier to justify. Others, such as neglecting the compliance of the brain parenchyma-which Romano et al.^17^ found to significantly affect peristaltic-induced flow-warrant further investigation , particularly in the context of aging-related reductions in brain stiffness. Studies using Magnetic Resonance Elastography have shown that brain stiffness decreases with age, with annual reductionsin the range of $-0.008$ to $-0.025$ kPa per year, leading to a softer, more deformable brain tissue^85^, which challenges our assumption of an undeformable parenchyma. Moreover, an increased parenchymal compliance could lead to greater local deformations in response to arterial pulsations, thereby reducing the effectiveness of our proposed pumping mechanism by diminishing cyclic changes in PVS width-one of the main drivers of our mechanism. Additionally, further research is needed to clarify how transmantle pressure evolves with age. While studies suggest an increase in peak transmantle pressure with age^86^, its precise effect on glymphatic flow remains uncertain, as it could either enhance or counteract perivascular fluid movement depending on the relative phase shift between transmantle pressure fluctuations and arterial pulsations. These factors underscore the need for further investigations into the interplay between arterial pulsations, transmantle pressure, and the progressive changes in brain stiffness over time.

In summary, our model provides a foundational framework for investigating how the temporal interplay between arterial pulsations and pressure fluctuations influences periarterial bulk flow. The results indicate that, under conditions within physiologically relevant ranges, the proposed mechanism can indeed drive bulk motion toward the brain’s interior, effectively counteracting the existing cycle-averaged pressure gradient. This insight not only advances our understanding of glymphatic flow dynamics but also highlights the potential of the hypothesized mechanism as a significant contributor to the clearance processes within the brain. While further refinements are required for accurate quantitative predictions, our findings underscore the importance of this mechanism in the context of glymphatic flow.

## Data Availability

The datasets generated during and/or analysed during the current study are available from the corresponding author on reasonable request.
